# Clinical Acceptability of the Internal Gap of CAD/CAM PD-AG Crowns Using Intraoral Digital Impressions

**DOI:** 10.1155/2016/7065454

**Published:** 2016-11-28

**Authors:** Tae-Gyung Kim, Sungtae Kim, Hyunmin Choi, Jae-Hoon Lee, Jae-Hong Kim, Hong-Seok Moon

**Affiliations:** ^1^Department of Prosthodontics, Yonsei University, College of Dentistry, 50 Yonseo-ro, Seodaemun-gu, Seoul 03722, Republic of Korea; ^2^Department of Periodontology, Dental Research Institute, Seoul National University, School of Dentistry, 101 Daehak-ro, Jongno-gu, Seoul 110-768, Republic of Korea; ^3^Department of Dental Technology, School of Medical and Public Health, Kyungdong University, 815 Gyeonhwon-ro, Wonju, Gangwondo 24695, Republic of Korea

## Abstract

The purpose of this study was to compare the internal gap between CAD/CAM palladium-silver crowns and cast gold crowns generated from intraoral digital versus conventional impressions and to determine the clinical acceptability. Nickel-chrome master dies were made from the prepared resin tooth with the conventional impression method (*n* = 40). For ICC (Intraoral, CAD/CAM) group, 10 intraoral digital impressions were made, and 10 CAD/CAM crowns of a PD-AG (palladium-silver) machinable alloy were generated. For IC (Intraoral, Cast) group, 10 gold crowns were cast from ten intraoral digital impressions. For CCC (Conventional, CAD/CAM) group, 10 CAD/CAM PD-AG crowns were made using the conventional impression method. For CC (Conventional, Cast) group, 10 gold crowns were fabricated from 10 conventional impressions. One hundred magnifications of the internal gaps of each crown were measured at 50 points with an optical microscope and these values were statistically analyzed using a two-way analysis of variance (*α* = 0.05). The internal gap of the intraoral digital impression group was significantly larger than in the conventional impression group (*P* < 0.05). No significant difference was observed between the CAD/CAM group and the cast group (*P* > 0.05). Within the limitations of this* in vitro* study, crowns from intraoral digital impressions showed larger internal gap values than crowns from conventional impressions.

## 1. Introduction

There are several problems with the conventional analog impression method, including high risk of cross-contamination and technical errors, requirements for several impression materials and laboratory processes, and patient's discomfort [1, 2]. In addition, approximately, one-third of dental clinicians remake impressions three or more times [3].

Recently, several intraoral digital impression devices have been developed, providing more convenient treatment than the conventional impression method. One of these is i-Tero digital impression device invented by Cadent in 2005. This electronic impression device scans dental arch and tooth structures while the patient is at chair-side using an oral scanner, with no impression material and tray and displays the 3-dimensional image on the LCD monitor and transmits it to the laboratory technician [2, 4, 5]. In this way, it provides the digital data necessary for fabricating accurate master models to a CAD/CAM machine [2]. In other words, i-Tero is an independent digital impression device not connected to an in-office milling machine other than the CEREC and E4D intraoral digital impression devices [3].

The i-Tero digital impression device has several advantages compared with other intraoral digital impression devices because it is based on a parallel confocal principle. Light through a small pinhole focuses on the target subject and then is reflected [4]. Light will be returned through the pinhole only by a subject at the proper focal length and can be recognized by an oral sensor. Points out of the confocal plane cannot pass light through the pinhole. By employing this principle, i-Tero does not require any scanning powder [3, 4]. It can also scan the dental arch where it makes contact with the teeth for use of telecentric aperture. The telecentric aperture only passes the rays of light parallel to the optical axis. It causes the i-Tero to keep the same field of view without concern regarding the distance from the subject. However, research on the accuracy of prostheses fabricated from i-Tero digital impressions remains relatively rare.

Currently, while the popularity of the CAD/CAM laboratory systems is increasing dramatically, initial CAD/CAM prostheses have been criticized for the poor fit by some authors [6–8]. However, this defect has been improved quickly and many recent studies reported that the fit of the CAD/CAM all-ceramic crowns is similar to that of conventionally fabricated crowns [6, 9–13].

Innovium is a new palladium-silver (Pd-Ag) alloy invented by Ceragem Biosys. This noble metal alloy can be manufactured using the CAD/CAM system. Until now, only a few alloys of prosthetic metals have been used in dental milling machines. There was a general disinclination toward use of noble metal alloys in the CAD/CAM system because of the high metal attrition and the high material costs [5]. The advantage of this alloy is that it is cost-effective (20% less precious metal content), time-saving, and biocompatible. It has a bright yellow color like gold but has less problems with casting shrinkage, corrosion, and discoloration than base metal dental alloys. But there is little research on the accuracy of CAD/CAM prostheses fabricated using this alloy (Innovium).

Close internal fit is one of the most important factors affecting the accuracy and the long-term success of the fixed prostheses [14–17]. Insufficient adaptation of prostheses can result in an increase in plaque accumulation, ultimately leading to periodontal diseases, secondary caries, and pulpal inflammation of abutment teeth. These are major causes of the prosthetic failure [18–22].

Therefore, the purpose of this study was to compare the internal gap between CAD/CAM Pd-Ag (Innovium) crowns and cast gold crowns generated from intraoral digital versus conventional impressions and to determine the clinical acceptability of the internal gap of CAD/CAM Pd-Ag (Innovium) crowns using intraoral digital impressions. The null hypothesis of this study is that there is no statistically significant difference in the internal gap between CAD/CAM Pd-Ag (Innovium) crowns and cast gold crowns generated from intraoral digital versus conventional impression.

## 2. Materials and Methods

A resin tooth of a mandibular right first molar in a typodont model (typodont model; Nissin Dental Products, Inc.) was used. It was prepared for a full veneer crown with a chamfer margin. The milling machine (D-F 44; Harnisch-Reith) was used to allow the model to have a total occlusal convergence angle of 12 degrees [5, 9, 23].

This resin tooth was duplicated with heavy-body and light-body addition-type silicone impression materials (Exafine; GC Corporation), mixed according to the manufacturer's recommendations, to make 40 impressions using perforated partial metal impression tray (GC impression tray; GC America Inc.). An autopolymerizing acrylic resin (pattern resin; GC Corporation) was poured into the impressions to form patterns that were used to fabricate metal master dies [9]. Forty acrylic resin patterns were invested and cast with a nickel-chrome alloy (Verabond 2; Aalbadent) to prevent abrasion and ensure the standardization of shape and dimension of specimens [9, 17]. For setting the respective crown to the corresponding master die in exactly the same position, pattern resin jigs were used.

**Figure 1:**
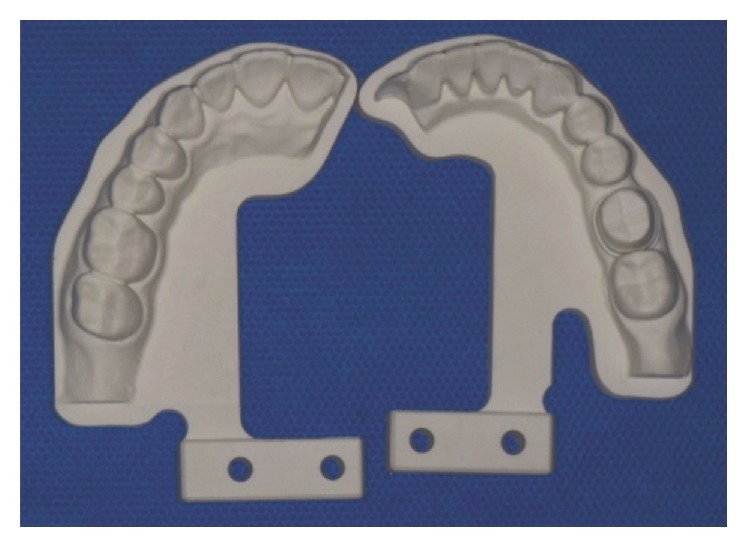
Polyurethane model made from intraoral digital impression.

Forty master dies were divided into four groups. For ICC group, 10 intraoral digital impressions (i-Tero; Cadent) were made, and 10 CAD/CAM crowns of a Pd-Ag machinable alloy (Innovium; Ceragem Biosys.) were generated. For IC group, 10 polyurethane models were made from 10 intraoral digital impressions and then gold crowns were fabricated on these models (Figure 1). For CCC group (*n* = 10), CAD/CAM Pd-Ag crowns were made using the conventional impression method. For CC group (*n* = 10), gold crowns were fabricated from 10 conventional impressions. When making conventional impressions, a one-step putty and wash impression technique was used and the type of impression tray used was perforated metal tray (GC impression tray; GC America Inc.). After applying the wetting agent on the inner surface of the acquired impression, a vacuum mixer was used to mix the Type IV dental stone (Fujirock EP, GC America Inc.) at a water-powder ratio specified by the manufacturer. The mix was carefully injected, making sure to avoid bubbles forming in the dental vibrator. The fabricated plaster molds were kept in a place where the temperature and humidity were kept constant. The luting space of CAD/CAM Pd-Ag crowns was set at 25 *μ*m. For fabrication of gold crowns, two coats of die spacer (Pico-Fit; Renfert) were applied on the casts. The mean thickness of the die spacer was expected to be about 25 *μ*m [24]. The wax pattern was created by the convention method of using a dipping wax (elaflex, Brident). The wax pattern used silicate-based casting investments (CB30, Ticonium), in accordance with the traditional lost wax technique. Type IV gold alloy (C-55, Shinhung dental gold alloy) was melted and then injected with the use of a high-frequency casting machine (Fornax, BEGO). All crowns were fabricated by only one dental technician to avoid discrepancies in the laboratory technique.

**Figure 2:**
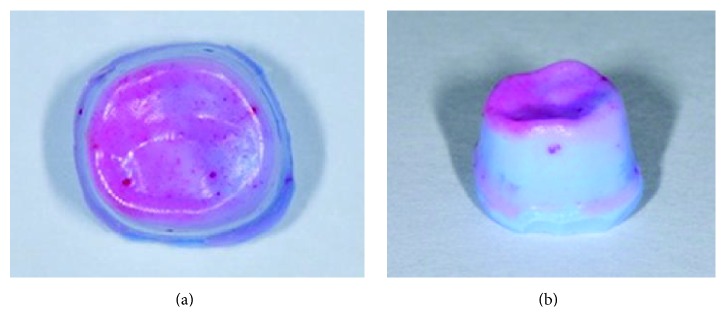
(a) Specimen using Fit Checker II (silicone film on the top of the light-body impression material): occlusal view. (b) Specimen using Fit Checker II (silicone film on the top of the light-body impression material): buccal view.

A white low-viscosity silicone material (Fit Checker II; GC Corporation) was mixed with a pink disclosing agent that made the material easily recognizable under an optical microscope. It was also ensured that a pink disclosing agent used was kept minimal (a drop) and the same amount was used each time. The mixture was applied to the inner surface of the crowns then the respective crown was seated on the corresponding metal master die with a pattern resin jig [23, 25]. A compressive force of 40 N was placed on the crowns using a universal testing machine (*EZ*-test; Shimadzu) for 5 minutes to simulate clinical cementation of the crown [23]. After polymerization, the master dies were removed, with the silicone film adhering to the inner surface of the crowns. To stabilize the film, a light-body addition-type silicone impression material (Exafine; GC Corporation) was injected into the crowns to form one piece with the silicone film. After setting, each specimen (a silicone film on the top of the light-body impression material) was carefully removed from the crowns (Figures 2(a) and 2(b)). Specimens were cut into four pieces, buccolingually and mesiodistally, using a sharp knife. One hundred magnifications of the internal gaps (silicone film thickness) of each crown were measured with an optical microscope (Axio Imager; Zeiss), calibrated with a reference micrometer slide according to manufacturer's recommendation. For the internal gaps, 15 points were established buccolingually in an area spanning 1 mm from the supraversional margin to 1 mm from the bottom of the occlusal surface. Mesiodistally, 10 points, spaced evenly, were selected between 0.5 mm from the supraversional margin to 0.5 mm from the bottom of the occlusal surface. Each experimental group was assigned 10 specimens; a single experimenter repeated the procedure to ensure that the points designated would be consistent, as much as possible, between each specimen. Fifty parameters per specimen were registered [9].

All 2000 points (50 points × 40 specimens) were recorded. Measured parameters were expressed as means and standard deviations (SD) for 4 groups. The data were statistically analyzed using a two-way analysis of variance (ANOVA) to find the effect of the impression and fabrication methods on the internal gap of crowns. Tukey's honest significant difference (HSD) test was used to compare the statistical differences between each group after verification. Statistical significance was set at *P* < 0.05. All statistical analyses were performed using SPSS 18.0 for Windows (SPSS Inc.).

## 3. Results

Table 1 shows the means ± SD of the internal gap of each group. Using ANOVA (*α* = 0.05), the effect of the impression and fabrication methods on the internal gap of the crowns was analyzed. A two-way ANOVA model using the main effect of impression and fabrication methods described 26.3% of the variation in internal gaps.

**Figure 3:**
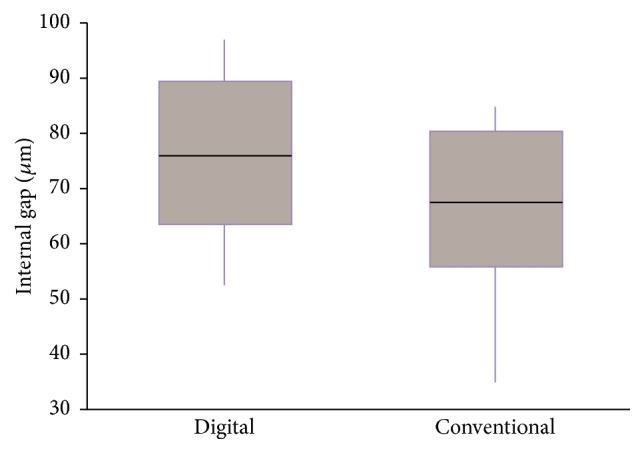
Box plot diagram of the internal gap comparing the two impression methods.

**Figure 4:**
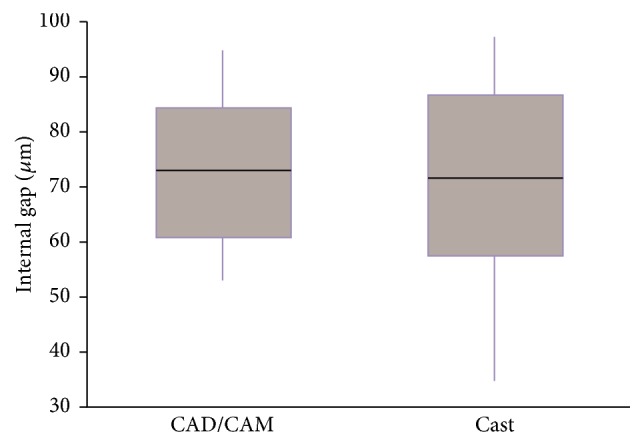
Box plot diagram of the internal gap comparing the two fabrication methods.

In a* post hoc* analysis by multiple comparison analysis, the two impression method groups showed a significant difference (*P* < 0.05). The mean internal gap value of the intraoral digital impression group was 76.7 ± 13.1 *μ*m, whereas the mean internal gap value of the conventional impression group was 68.1 ± 12.9 *μ*m (Figure 3). The internal gap of the intraoral digital impression group was significantly larger than in the conventional impression group. However, the internal gap of the CAD/CAM group (72.7 ± 12.3 *μ*m) and the cast group (72.0 ± 15.1 *μ*m) did not differ significantly from each other (*P* > 0.05) (Figure 4).

## 4. Discussion

The results of this study support rejecting the null hypothesis. In the present study, the mean ± SD internal gaps for the intraoral digital and conventional impression groups were 76.7 ± 13.1 *μ*m and 68.1 ± 12.9 *μ*m, respectively. Crowns from intraoral digital impressions showed significantly larger internal gap values than crowns from conventional impressions. This difference may be attributed to technical mistakes by the operator because the intraoral digital impression device requires a learning and adaptation process for beginners [5, 26]. The second possible factor influencing the outcome is the relatively low precision of the oral scanner compared with the conventional impression materials. Currently, there is little research on the precision of the i-Tero scanner. However, on the CEREC system, several authors report that the CEREC camera creates a shadow distal to a target object, and this distal shadow phenomenon could adversely affect the internal gap of CEREC restorations [27].

There was no significant difference in internal gap between CAD/CAM Pd-Ag crowns (72.7 ± 12.3 *μ*m) and cast gold crowns (72.0 ± 15.1 *μ*m). In other words, the internal gap of CAD/CAM palladium-silver crowns was similar to that of cast gold crowns. These results are in agreement with other authors who report that CAD/CAM ceramic crowns had the same or better accuracy in the gap as conventional ceramic crowns [6, 9–13, 28]. However, previous studies are in contrast to this result reporting that cast titanium crowns showed better marginal fit than the CAD/CAM titanium crowns [29, 30].

According to McLean and von Fraunhofer, the clinically acceptable limit of gaps in the restoration is 120 *μ*m. The means of internal gaps of all groups in this study were within 120 *μ*m [31]. Therefore, crowns showed clinically acceptable internal gap regardless of the impression and fabrication methods. This result supports the clinical utilization of the intraoral digital impression device and the CAD/CAM Pd-Ag (Innovium) crowns.

The use of the intraoral digital impression devices and the CAD/CAM system will give us many benefits. They can help clinicians save time and reduce many labor-intensive laboratory procedures. Also, they can provide better patient's comfort [26]. Within this study, they showed clinically acceptable results on the internal gap of crowns.

The machinable Pd-Ag alloy (Innovium) has several advantages. It is cost-effective, biocompatible, and requires no model in the CAD/CAM procedure. In the present study, CAD/CAM Pd-Ag crowns showed similar internal gap compared with cast gold crowns. Therefore, this alloy may be considered as an alternative to gold.

However, this study has methodological limitations. To obtain the internal gap values of crowns, various methods have been suggested to measure the internal gap of prostheses [9, 15, 32, 33]. Grenade et al. sectioned all die/coping sets to directly measure the thickness of the cement [15]. This method can provide more accurate parameters than the method used to create silicone replicas because the replica technique can have defects in the silicone material at the point of measurement [32]. Ucar et al. weighed the light-body addition silicone to get data on the three-dimensional volumes between the crowns and their dies [33]. Lee et al. calculated the internal gap using the formula (silicone film thickness = weight/surface area × density) because they considered that using limited measuring points was not accurate [9]. In this study, internal gaps were registered by measuring the silicone film thickness on sectioned specimens. This displayed only two-dimensional information in the cutting plane [33] and the internal gap width was represented by restricted 50 points per specimen.

In addition to the internal gap, there are many other factors involved in evaluating the accuracy of fixed prostheses such as marginal fit, interproximal contact quality, and occlusion. Also, this study was performed* in vitro* with only a single crown. More comparative experimental studies under real clinical conditions, with long-span prostheses, and on other factors are needed.

## 5. Conclusion

Within the limitations of this* in vitro* study, the following conclusions were drawn:

1. Crowns from intraoral digital impressions showed larger internal gap values than crowns from conventional impressions.
2. The internal gap of CAD/CAM Pd-Ag crowns was similar to that of cast gold crowns.
3. All crowns showed clinically acceptable internal gap, regardless of the impression and fabrication methods.

## Figures and Tables

**Table 1:** Comparison of mean internal gap by two types of impression methods and by two types of fabrication methods (*μ*m).

Fabrication	Impression	
	Mean (±SD)^*∗*^	
	Intraoral digital	Conventional
CAD/CAM	77.7 (±12.0)^Aa#^	67.7 (±11.0)^Bb^
Cast	75.6 (±14.8)^Aa^	68.4 (±15.2)^Bb^

^*∗*^Means and standard deviations in parentheses.

^#^Data with the different letters are significantly different at 0.05 significance level.

Uppercased letters mean the comparison in the types of impression method and lowercased letters mean the comparison in the types of fabrication method.
